# ISGylation: is our genome yearning for such a modification? ISGylation function & implication in health

**DOI:** 10.3724/abbs.2025028

**Published:** 2025-02-28

**Authors:** Zheng Chen, Zheng Li, Ying Wang, Zaure Dushimova, Kapanova Gulnara, Shunichi Takeda, Zhongjun Zhou, Xingzhi Xu

**Affiliations:** 1 Shenzhen University General Hospital-Dehua Hospital Joint Research Center on Precision Medicine (sgh-dhhCPM) Dehua Hospital Dehua 362500 China; 2 State Key Laboratory of Agrobiotechnology and MOA Key Laboratory of Soil Microbiology College of Biological Sciences China Agricultural University Beijing 100193 China; 3 Guangdong Key Laboratory for Genome Stability & Disease Prevention and Carson International Cancer Center Marshall Laboratory of Biomedical Engineering Shenzhen University Medical School Shenzhen 518060 China; 4Al-Farabi Kazakh National University 71 Al-Farabi Avenue Almaty 050040 Kazakhstan; 5 School of Biomedical Sciences University of Hong Kong Hong Kong 999077 China

**Keywords:** antibacterial immunity, antiviral immunity, genomic stability, ISG15, ISGylation

## Abstract

ISGylation is the post-translational modification of protein substrates covalently conjugated with the ubiquitin-like protein, interferon-stimulated gene 15 (ISG15). Although initially linked to antiviral immunity, recent evidence highlights important roles for ISGylation in various biological processes, such as maintaining genomic stability, promoting tumourigenesis, and being involved in other pathological conditions. In this review, we examine the molecular mechanisms underlying ISGylation, its interplay with other post-translational modifications, and its involvement in diverse biological and pathological processes. We propose future research directions to advance the field and discuss how ISGylation might be harnessed to ensure human health, particularly genome instability-associated diseases.

## Introduction

**Figure 1:**
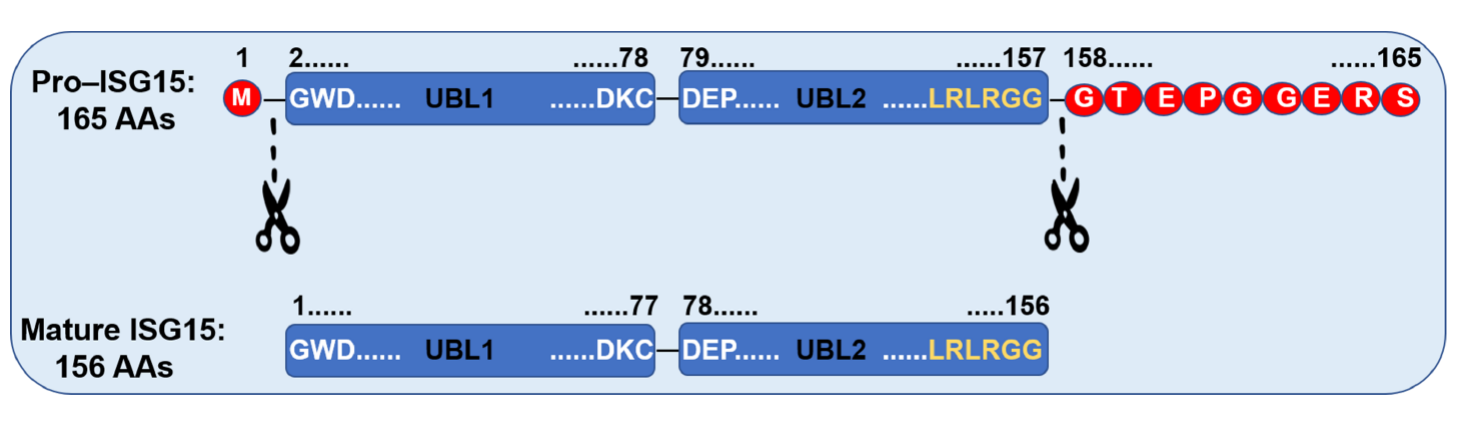
Figure 1 The functional maturation of ISG15 Mature ISG15 is generated through the removal of the N-terminal methionine and the final eight amino acids (AAs) of the C terminus. The N-terminal UBL1 domain (1–77 AAs) starting with Glycine (G) and ending with Cystine (C) is critical for the E3 ligase-mediated transfer of ISG15 from the E2 enzyme to a lysine residue on the target protein, while the C-terminal UBL2 domain starting with Aspartic acid (D) and end with Glycine (G) facilitates ISG15 conjugation to E1 and E2 enzymes during ISGylation. The LRLRGG motif is responsible for substrate targeting.

Host cells release interferons (IFNs) in response to foreign pathogens or abnormal endogenous signals. These IFNs bind to the interferon-stimulated response element (ISRE) on specific promoters, thereby activating the expression of interferon-stimulated genes (ISGs) [ 1, 2]. Among these, ISG15 is a 15 kDa protein initially discovered in IFN-treated Ehrlich ascites tumor (EAT) cells [3] and was previously referred to as ubiquitin cross-reactive protein (UCRP) [4]. ISG15 is typically expressed at low levels under physiological conditions but is one of the most highly expressed ISGs when activated by type I interferons (IFN-Is), lipopolysaccharide (LPS), DNA damage, viral and bacterial infections, or other pathogenic stimuli [ 1, 5, 6]. ISG15 comprises two tandem repeats of ubiquitin-like (UBL) domains, each playing distinct roles in ISGylation [ 7, 8]. It was also identified as the first member of the Class 1 UBL proteins superfamily [ 9, 10]. Initially synthesized as a 165-amino-acid precursor, pro-ISG15 undergoes processing by a human ortholog of yeast ubiquitin-specific protease Ubp1 or a related protein ( Figure 1) [10]. This protease specifically cleaves the Gly157-Gly158 peptide bond, in addition to the removal of NH2-terminal methionine to yield a mature ISG15 protein of 156 amino acids [ 9, 10]. This maturation process is essential for ISG15’s functionality within the ubiquitin (Ub)-like modification system. A conserved motif consisting of lysine, arginine, and glycine residues (151-LRLRGG-156) at the end of the C-terminal is vital for ISG15’s binding to specific target proteins by sequence similarity [ 8, 11, 12].

**Figure 2:**
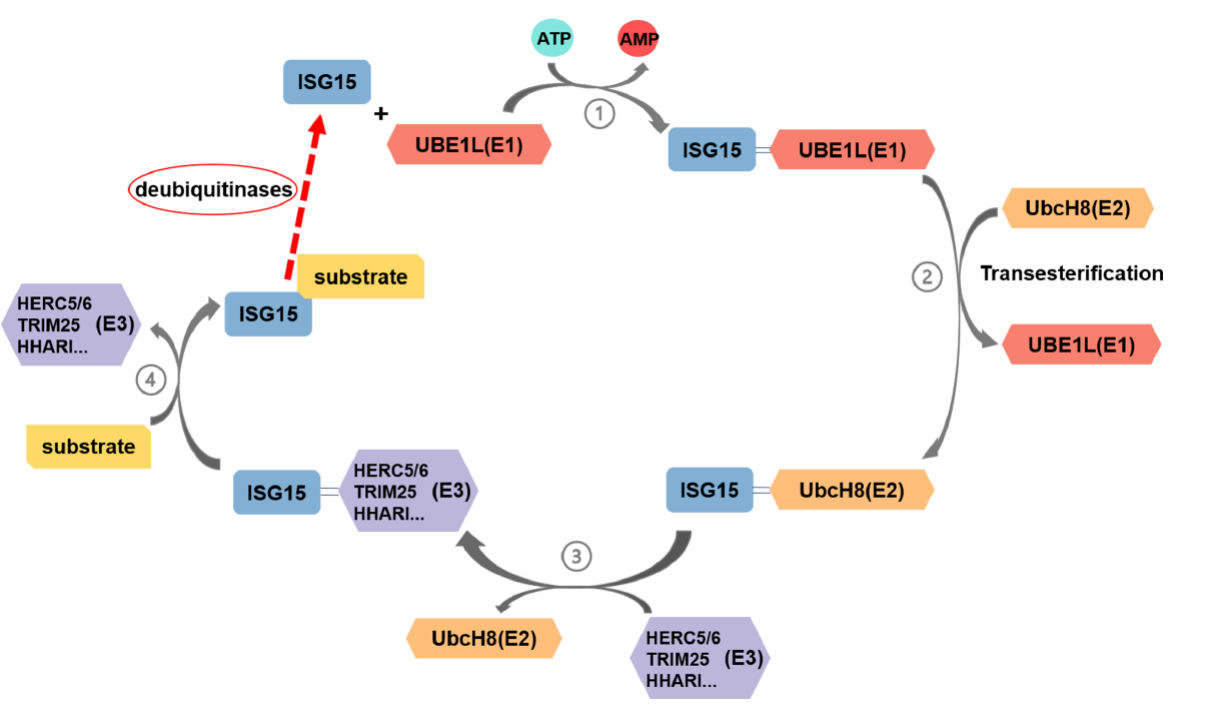
Figure 2 ISGylation ISGylation occurs in four steps: (1) ISG15 binds to the E1 enzyme in an ATP-dependent manner; (2) ISG15 is transferred from the E1 to the E2 enzyme via transesterification; (3) the E2-ISG15 complex recruits an E3 ligase and transfers ISG15 to the E3 enzyme; and (4) the E3-ISG15 complex targets a substrate, facilitating the conjugation of ISG15 to the substrate. The conjugation of ISG15 and substrate will be de-ISGylated by deubiquitinases.

The process of ISGylation ( Figure 2) involves a series of enzymatic steps analogous to ubiquitination. First, the E1-activating enzyme, such as Ub-like modifier-activating enzyme 7 (UBA7, also known as UBE1L), binds to ISG15’s C terminus via a high-energy thioester bond, facilitated by the hydrolysis of ATP [13]. Next, active ISG15 is transferred to the E2-conjugating enzyme, such as ubiquitin-conjugating enzyme E2 UbcH8 (also known as UBE2E2, the primary E2 enzyme for ISG15 conjugation) [ 14, 15], or UbcH6 (also known as UBE2E1) [16], through a transesterification reaction. These steps rely on the UBL2 domain of ISG15. Subsequently, UbcH8 transfers ISG15 to E3 ligases such as HECT and RLD domain contained E3 ubiquitin-protein ligase 5/6 (HERC5/6) [ 17– 20], tripartite motif containing 25 (TRIM25) [21], SCF (Skp1-Cul1-F-box) protein E3 ligase SCF ^FBXL19^ [22], human homolog of Ariadne (HHARI) [23], and DTX3L [24]. Finally, E3 ligases catalyze the covalent attachment of ISG15 to target proteins on lysine residues ( Table 1), a process facilitated by the UBL1 domain of ISG15.

**Table:** **Table 1** Known E1/E2/E3 and substrates of ISGylation

E1s	E2s	E3s	Substrates
UBE1L	UbcH6/UbcH8	hHERC5/mHERC6	Parkin K349/369 [19]
			STAT1 [ 25– 27]
			STING K150 [20]
			K224/236/289/345/370 [28]
			IRF3 K193/360/366 [29]
			cGAS K21/187/219/458 [30]
			NLRP3 K799 [31]
			P53 [ 32– 35]
			PTEN [36]
			tAIF [37]
		DTX3L	LIPG [24]
		TRIM25	14-3-3σ [21]
			HAhnRNPA2B1 K22 [38]
		SCF ^FBXL19^	NF-κBp65 [22]
		HHARI	4EHP [23]
		Undefined now*	Ub K29/48 [39]
			UbcH6 K136 [16]
			UbcH13 K92 [40]
			KPNA2 [41]
			EMD K37 [42]
			JAK1 [43]
			RIG-1 [44]
			PD-L1 [45]
			RARα [46]
			CHIP K143/144/145 [47]
			HIF-1α [48]
			TSG101 [ 49, 50]
			FOXO3a [51]
			FLNC [52]
			ADAMTS1 K309/593/597/602 [53]
			β-catenin [54]
			PKR K69/159 [55]
			mTOR [56]
			RBM47 K329 [57]
			Spi2A [58]
			MDA5 K23/43 [59]
			NS1A K41 [60]
			YAP [61]
			SIRT1 [62]
			DRP1 [63]
			CPS1 [64]
			NOX4 [65]

*“Undefined now” means that the E3 ligase has not been identified.

Similar to ubiquitination, ISGylation is reversible due to the activity of deubiquitinases (DUBs) via a mechanism referred to as deISGylation. We will discuss this intriguing overlap between ISGylation and ubiquitination in the next section. Ubiquitin-specific peptidase 18 (USP18, also known as UBP43) is the primary ISG15-specific dUB that removes ISG15 from target proteins [ 13, 66, 67]. In addition, other members of the USP protease family, including USP2, USP5, USP13, USP14, USP16, and USP21, have been identified as ISG15-reactive proteases with similar functionalities [ 68– 70].

In this review, we will discuss the relationship between ISGylation and other cellular post-translational modifications, as well as its function in immune response, genome stability, tumor development and human health, to provide new ideas for future functional research.

## Relationship between ISGylation and Other Post-translational Modifications (PTMs)

Protein post-translational modification is an epigenetic mechanism that covalently adds or removes functional groups to amino acid residues of proteins to regulate protein stability, activity, localization, and interaction with cellular molecules such as proteins and nucleic acids. These modifications include phosphorylation, ubiquitination, SUMOylation, glycosylation, methylation, acetylation, lipidation, and protein hydrolysis, affecting almost all aspects of normal cell biology and pathogenesis. Here, we would like to discuss the crosstalk between ISGylation and different post-translational modifications such as phosphorylation, ubiquitination, and SUMOylation.

### ISGylation and ubiquitination

**Figure 3:**
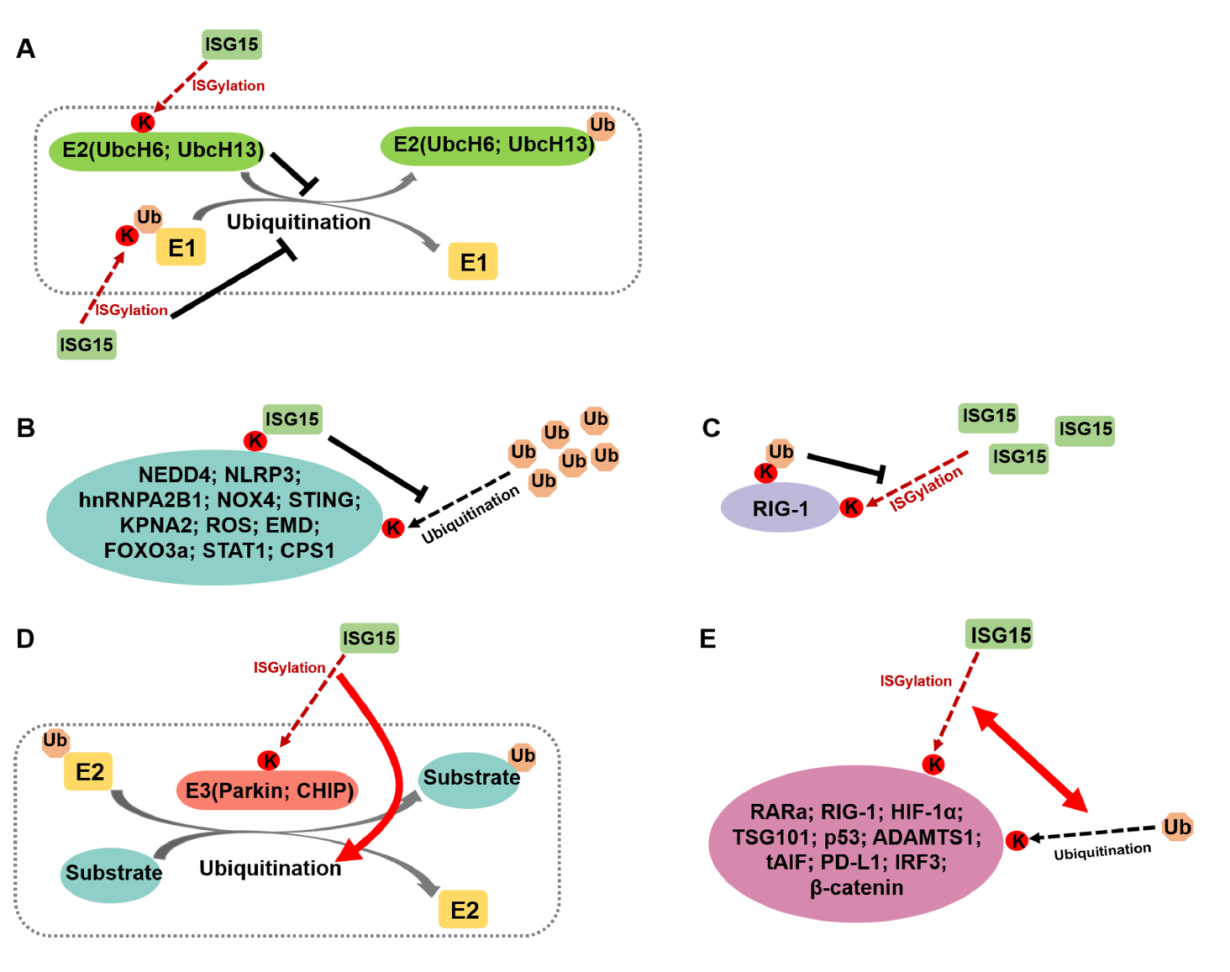
Figure 3 Crosstalk between ISGylation and ubiquitination (A) ISGylation of E2 enzymes (UbcH6, UbcH13) and Ub compromise their ubiquitination activity. (B) ISGylation of other proteins similarly inhibits their ubiquitination. (C) RIG-I ubiquitination suppresses its own ISGylation. (D) ISGylation of E3 ligase (Parkin, CHIP) antagonizes their ubiquitination activity. (E) ISGylation of other proteins has antagonistic effects on their ubiquitination. K: Lys; Ub: ubiquitin; the red dashed line: ISGylation; the black dashed line: ubiquitination; the solid black line: inhibition; the solid red arrow: promotion.

ISGylation and ubiquitination primarily modify lysine residues, enabling different types of modifications to probably occur at the same Lys site or influence each other. This interplay between ISGylation and ubiquitination establishes complex regulatory mechanisms that finely control various aspects of protein biology, including protein function, localization, stability, and broader cellular processes [71]. The dynamic nature of these modifications allows cells to respond effectively to diverse stimuli and stress conditions, highlighting their pivotal roles in development, signal transduction, and the maintenance of cellular homeostasis [72]. The relationship between ubiquitination and ISGylation involves a nuanced interplay of antagonistic and synergistic interactions, depending on the specific substrates undergoing modification ( Figure 3).

In most cases, ISGylation and ubiquitination antagonize each other. ISGylation can stabilize proteins by antagonizing ubiquitination-mediated proteasomal degradation. Indeed, in cells with Ataxia Telangiectasia (A-T) or ATM deficiency, elevated ISG15 expression coincides with impaired proteasome-mediated protein degradation, suggesting that ISG15 conjugation interferes with this process [73]. Ub itself is a substrate for ISGylation [39]. ISGylation at Lys29 of Ub forms an ISG15-Ub mixed chain on substrates, which disrupts the homeostasis of ubiquitinated proteins [39]. Furthermore, siRNA-mediated knockdown of *ISG15* and *UbcH8* (the primary E2 enzyme for ISG15) or acetaldehyde (Ach) treatment, which induces USP18 expression (a deISGylation protease), leads to an upregulation of polyubiquitinated proteins, further supporting the antagonistic relationship between ISGylation and ubiquitination [ 26, 74].

ISGylation can also impair the catalytic activity of ubiquitination-related enzymes. For instance, ISGylation at Lys92 of UbcH13 and Lys136 of UbcH6 reduces their ability to conjugate Ub [ 16, 75]. Similarly, ISGylation of the E3 ligase neuronally expressed developmentally downregulated 4 (NEDD4), which ubiquitinates retroviral group-specific antigen precursors and matrix proteins to facilitate virus release [76], disrupts its interaction with E2 enzymes. This action prevents the ubiquitination of the Ebola virus matrix protein VP40, enhancing the innate antiviral response [ 77, 78], which we discuss in more detail later.

In addition to disrupting the ubiquitination machinery, ISGylation can antagonize the ubiquitination of substrates. For example, ISGylation of NOD-, LRR-, and pyrin domain-containing protein 3 (NLRP3) by HERCs suppresses K48-linked ubiquitination, thereby inhibiting NLRP3 inflammasome activation [31]. Similarly, ISGylation of heterogeneous nuclear ribonucleoprotein A2/B1 (hnRNPA2B1) mediated by prostate cancer associated transcript 6 (PCAT6) protects hnRNPA2B1 from ubiquitin-dependent degradation [38]. Other substrates, such as NADPH oxidase 4 (NOX4), stimulator of interferon genes (STING), karyopherin subunit alpha 2 (KPNA2), skeletal protein Emerin (EMD), carbamoyl phosphate synthetase-1 (CPS1), and FOXO3a, are also protected from ubiquitination-dependent degradation through ISGylation [ 20, 41, 42, 51, 64, 65].

Conversely, ubiquitination can negatively regulate ISGylation. For example, the human cytomegalovirus (HCMV) UL50-encoded transmembrane protein pUL50 facilitates the ubiquitination of UBE1L (the E1 enzyme for ISGylation) through RNF170 (an E3 ligase for ubiquitination), leading to its proteasomal degradation [79]. This complex interplay between ubiquitination and ISGylation reflects the regulatory mechanisms that govern cellular responses to stress and infection.

Besides the antagonistic effects, in some cases ISGylation and ubiquitination serve as positive feedback to each other. ISG15 and Ub can also synergize with each other. For instance, ISGylation of the ubiquitin E3 ligase Parkin at K349/369 and the carboxyl terminus of Hsp70-interacting protein (CHIP) at K143/144/145 enhances their E3 ligase activity [ 19, 47]. Similarly, ISGylation of a disintegrin and metalloproteinase with thrombospondin motifs 1 (ADAMTS1), programmed cell death ligand 1 (PD-L1), and truncated apoptosis-inducing factor (tAIF) promote their ubiquitination and subsequent proteasome-dependent degradation [ 37, 45, 53]. Such interactions illustrate the intricate balance between ISGylation and ubiquitination in cellular processes.

### ISGylation and phosphorylation

**Figure 4:**
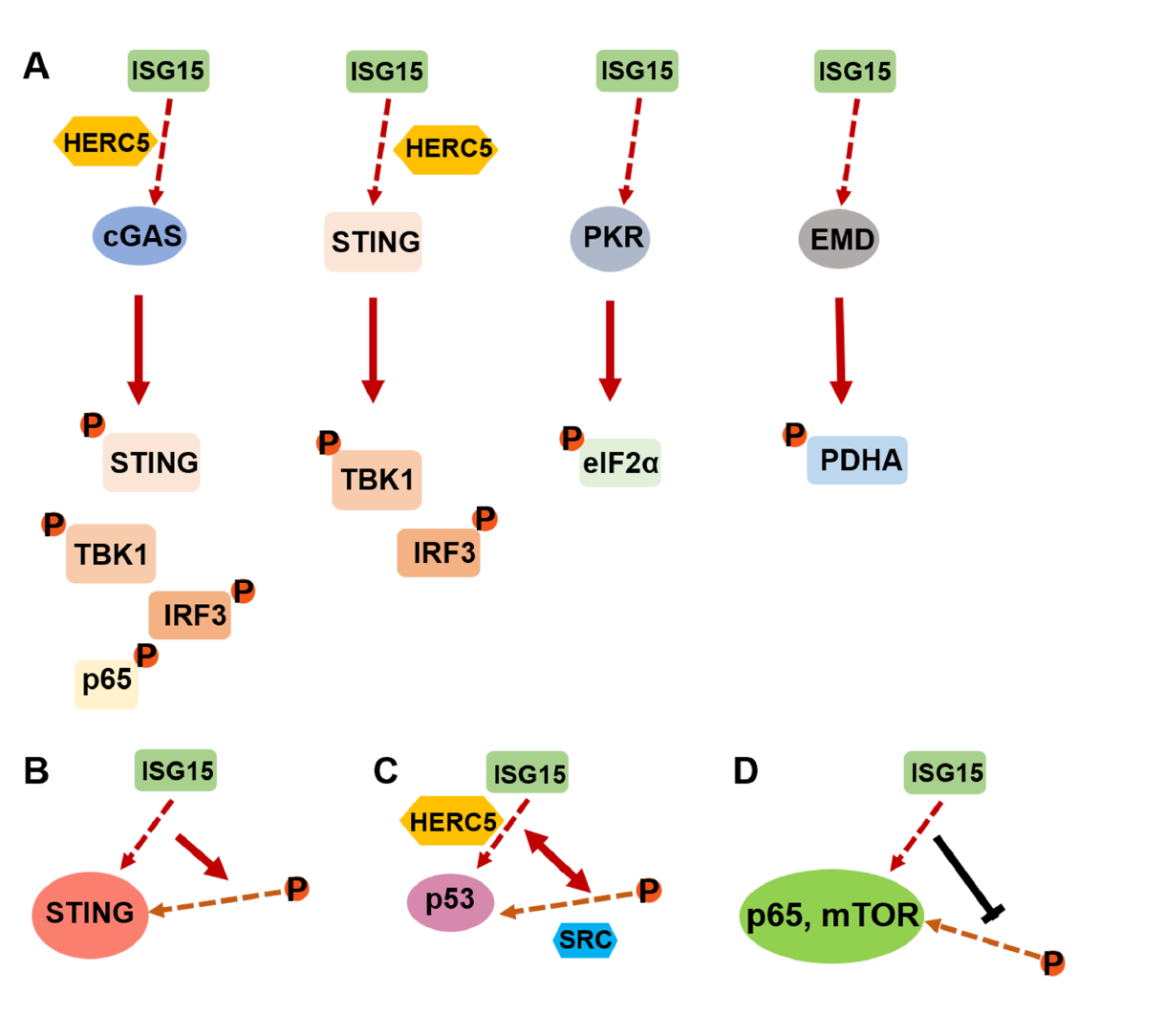
Figure 4 Crosstalk between ISGylation and phosphorylation (A) HERC5-mediated cGAS and STING ISGylation and ISGylation of PKR and EMD promote their downstream proteins phosphorylation. (B) STING ISGylation facilities its phosphorylation. (C) HECR5-mediated p53 ISGylation and SRC-mediated p53 phosphorylation antagonize each other. (D) ISGylation of p65 and mTOR suppress their phosphorylation. P: phosphate group; the red dashed line: ISGylation; the orange dashed line: phosphorylation; the solid red arrow: promotion; the solid black line: inhibition.

Phosphorylation is a reversible post-translational modification involving the addition of a phosphate group to serine, threonine, or tyrosine residues by kinases. This modification is essential in regulating numerous cellular processes such as signal transduction, protein-protein interactions, and enzyme activity [80]. Research suggests that these modifications can influence each other; for instance, phosphorylation may enhance or inhibit ISGylation by altering the accessibility of target proteins or the activity of the ISGylation machinery. Conversely, ISGylation can affect phosphorylation-related pathways by stabilizing or destabilizing phosphoproteins, thereby influencing various signaling cascades. This interplay highlights a complex regulatory network that fine-tunes cellular responses to environmental stimuli, stress, and immune challenges ( Figure 4).

#### ISGylation positively regulates phosphorylation

Protein ISGylation could activate downstream factors phosphorylation. For example, cGAS ISGylation promotes phosphorylation of STING, TANK binding kinase 1 (TBK1), IRF3, and the nuclear factor kappa B (NF-κB) subunit p65. HERC5 catalyzes the ISGylation of cyclic GMP-AMP synthase (cGAS), enhancing downstream signaling through cGAMP-mediated activation of STING, TBK1, IRF3, and the phosphorylation of p65 upon exogenous DNA transfection [30]. Silencing *USP18* not only elevates signal transducer and activator of transcription 1 (STAT1) phosphorylation but also enhances ISGylation levels [25]. Depletion of USP18 increases ISGylation, prolonging the phosphorylation of STAT1/2 and enhancing IFN signaling transduction [25]. Furthermore, the ISGylation of double-stranded RNA-dependent protein kinase (PKR) at K69/159 promotes the phosphorylation of the α-subunit of eukaryotic initiation factor 2 (eIF2α) [55]. Similarly, ISGylation of emerin (EMD) promotes its interaction with the catalytic alpha subunit of PDHc (PDHA) and facilitates PDHA S293/300 phosphorylation [42]. Some proteins’ ISGylation also improves their own phosphorylation. DNA damage-induced ISGylation of p53 facilitates its phosphorylation [32]. The SARS-CoV-2 protein PLpro, which exhibits deISGylation activity, removes HERC5-mediated ISGylation of STING, thereby inhibiting phosphorylation of STING and downstream TBK1/IRF3 [81].

#### ISGylation negatively regulates phosphorylation

ISGylation can also antagonize protein phosphorylation. For instance, ISGylation of NF-κB p65, mediated by the Skp1-Cul1-F-box (SCF) E3 ligase complex SCFFBXL19, inhibits p65 phosphorylation [22]. Similarly, ISGylation of mTOR may disrupt its phosphorylation. In ATP-binding cassette subfamily G member 2 (ABCG2)-deficient mice, mTOR remains inactive despite elevated ISGylation levels, which means promoting ISGylation does not facilitate mTOR phosphorylation [56].

Phosphorylation also can enhance ISGylation on phosphorylated substrates. For example, in transformed cells, SRC-mediated phosphorylation of p53 facilitates HERC5-mediated ISGylation, promoting p53 degradation and tumor progression [33]. Similarly, HERC5 shows preferential ISGylation of the phosphorylated N protein of SARS-CoV-2 [81]. Additionally, epinephrine-induced phosphorylation of the RNA-binding protein RBM47 at Ser309 primes it for ISGylation [57]. Based on the above findings, the relationship between ISGylation and ubiquitination is intricate and requires more research to explore.

### ISGylation and SUMOylation

SUMOylation is a post-translational modification in which Small Ubiquitin-like Modifier (SUMO) proteins are covalently attached to lysine residues on target proteins [82]. This process involves E1, E2 and E3 enzymatic cascades. Similar to ISGylation and ubiquitination, SUMOylation regulates various cellular processes by influencing the activity, stability, localization, or interactions of target proteins [ 82, 83]. The relationship between ISGylation and SUMOylation has been minimally explored; however, one study showed that the stable expression of SUMO3, but not SUMO1, enhanced overall ISGylation and ubiquitination levels in IFN-α-induced cells. This effect was evidenced by the increased stability of proteins such as UbcH8, HERC5, and TRIM25 [84]. To elucidate the regulatory relationship between ISGylation and SUMOylation, further mechanistic studies are needed.

## ISGylation in Mediating the Immune Response

During viral infections, host cells increase IFN-α/β levels, thus enhancing ISG15 synthesis in both infected and neighboring cells [ 85, 86]. IFN-β specifically stimulates ISG15 expression and promotes its secretion in macrophages [ 85, 87], while LPS boosts ISG15 conjugation and USP43 expression in these cells [5]. ISG15 is synthesized by many cell types, including human monocytes, fibroblasts, neutrophils, and lymphocytes, and exists in both free and conjugated forms [ 85, 88]. Free ISG15 modulates human immune responses by stimulating cytokine secretion and activating various immune cells; it plays a notable role in cytokine production and immune cell activation, supporting both innate and adaptive immunity [89]. For instance, co-incubation of ISG15 with peripheral blood mononuclear cells (PBMCs) or purified CD3 ^+^ cells induces IFN-γ [90]. Moreover, ISG15 promotes the proliferation of natural killer (NK) cells when co-cultured with T cells, which restricts target cell lysis, underscoring the importance of ISG15ylation stimulated by IFN-γ in this process [85]. Recombinant mouse ISG15 exhibits neutrophil chemotactic activity similar to natural IP17 (a neutrophil chemotactic, ~17 kDa), helping to recruit neutrophils to inflamed sites [91]. While free extracellular ISG15 is pivotal for the IFN-γ-mediated antibacterial response, intracellular ISG15 inhibits IFN-α/β signal transduction when bound to USP18 [92] This mechanism enhances the antiviral capabilities of human, but not murine, fibroblasts.

Conjugation by ISG15 promotes immune activation and suppresses inflammatory responses via ISGylation. For instance, the serine protease inhibitor 2A (Spi2A) was the first protein reported to undergo ISGylation following bacillus Calmette-Guérin infection [58]. Several immune-regulating factors, including IRF3, RIG-I, melanoma differentiation-associated protein 5 (MDA5), STAT1, and Janus kinase 1 (JAK1), are ISGylation substrates. IRF3 and MDA5 ISGylation are required for their activation in immune signal transduction. HERC5 catalyzes IRF3 ISGylation at Lys193/360/366, weakening its interaction with peptidylprolyl cis/trans isomerase NIMA-interacting 1 (PIN1) and sustaining IRF3 activation [29]. Similarly, ISGylation of MDA5 at Lys23/43 is essential for the antiviral response via the IFN signaling pathway [59]. USP18 upregulation compromises HCV-induced STAT1 phosphorylation, indicating that ISGylation supports IFN-α signaling [26]. ISGylation suppresses inflammation. In endothelial cells (ECs), SCFFBXL19-mediated ISGylation of NF-κB p65 inhibits inflammation [22]. ISGylation of the RNA-binding protein RBM47 at K329, dependent on its phosphorylation at Ser309, regulates immune activation and maintains lung homeostasis [57].

Interestingly, while ISGylation is known for its anti-inflammatory effects, it can also exacerbate inflammatory responses. For instance, ISGylation of RIG-1 suppresses basal and viral-induced IFN activation [44]. Meanwhile, in a mouse model of colitis-associated colon cancer, ISGylation has been implicated in exacerbating inflammation [93]. Elevated ISGylation levels of MX Dynamin Like GTPase 1 (MX-1), a selective substrate of IFN-α/β, have been observed in symptomatic COVID-19 patients but not in asymptomatic or uninfected individuals, suggesting a role in cytokine storms and inflammation [93]. Moreover, rotavirus (RV) infection increases free ISG15 expression while suppressing protein ISGylation [94]. This suppression is associated with UBE1L depletion caused by Ub-mediated degradation of UBE1L during RV infection progression [94]. These pieces of evidence demonstrated that ISGylation can also accelerate inflammatory responses.

### ISGylation plays a dual role in viral infection

The innate immune response is the first line of defense against viral invasion, recognizing virus-specific components, such as viral proteins, to detect infection. Infected cells release signaling molecules, including IFNs, which activate surrounding immune cells, such as T cells and natural killer cells, to target and eliminate the infected cells [95]. B cells produce antibodies that neutralize the virus and prevent its entry into healthy cells, while macrophages and other phagocytes engulf and digest virus-infected cells and viral particles. Infected cells may also limit viral spread through programmed cell death (apoptosis). Together, these pathways effectively eliminate viral infections [ 96, 97].

#### ISGylation antagonizes viral infection by inhibiting virus replication, reducing lethality, and promoting an anti-viral immune response

ISG15 enhances the antiviral capabilities of cells primarily through ISGylation. In some cases, ISGylation suppresses viral RNA replication and proliferation while promoting viral elimination. For example, lymphocytic choriomeningitis virus (LCMV) and vesicular stomatitis virus (VSV) can cause severe conditions such as lymphocytic choriomeningitis and myeloencephalitis, which are fatal in mice [98]. Mice depleted of USP18 are resistant to these severe inflammatory conditions, which correlates with reduced LCMV RNA replication and increased protein ISGylation [98]. Overexpression of ISG15 or the E1/E2 enzymes UBE1L and UbcH8 in 293T cells inhibits the budding of Ebola virus VP40 VLPs [78]. Additionally, overexpression of murine ISG15 (mISG15), but not a mutant form (mISG15 K151A) that disrupts target protein ISGylation, reduces Sindbis virus replication in IFN-α/β receptor-deficient mice and protects them from lethality following infection [99]. ISG15 conjugation strongly attenuates influenza A virus replication early in infection in human cells [100]. Mechanistically, the ISGylation of the NS1 protein of influenza A virus (NS1A) at Lys41 disrupts its interaction with importin-α, thereby restricting viral replication and promoting an antiviral response via IFN-β [60]. Similarly, ISGylation of STING enhances the type I interferon response induced by herpes simplex virus 1 (HSV-1) infection and inhibits viral replication [20]. Furthermore, the downregulation of USP18 accelerates the elimination of the hepatitis B virus (HBV) [101].

Free ISG15 also impairs the replication of human immunodeficiency virus type 1 (HIV-1) and inhibits virion release, with this effect reversible through siRNA-mediated ISG15 depletion [102]. Similarly, free ISG15 enhances host defenses against chikungunya virus (CHIKV) in neonatal mice, as demonstrated by the lack of ISGylation when UBE1L is inhibited [103]. Furthermore, USP18 stabilized by ISG15 counteracts the NS5-mediated degradation of signal transducer and activator of transcription 2 (STAT2), thereby limiting the replicative efficacy of Dengue Virus (DV) and Zika Virus (ZIKA), even though ISGylation does not seem to be involved in these processes [104].

ISG15 also reduces lethality associated with Sindbis virus infection and suppresses Sindbis virus replication across multiple organs, an effect dependent on the LRLRGG motif for ISG15 conjugation to intracellular target proteins [105]. Furthermore, ISG15-mediated ISGylation protects murine cardiomyocytes from Coxsackievirus B3 (CVB3) infection [106]. Finally, ISGylation can protect mice from lethality caused by Influenza A and Sendai viruses without impacting virus replication or the immune response [107].

ISGylation also plays a pivotal role in promoting anti-viral immunity. Early studies showed that ISG15-deficient mice are more susceptible to influenza A/WSN/33, influenza B/Lee/40, hepatitis A virus type 1 (HAV-1), and murine gamma herpes virus infections [108]. In human cells, siRNA-mediated downregulation of ISG15 conjugation enzymes significantly restricted the antiviral response against the Influenza A virus [100]. ISGylation also enhances resistance to human respiratory syncytial virus (RSV) [109]. Furthermore, ISGylation of NLPR3 stabilizes the protein and enhances inflammasome activation in response to SARS-COV-2 [31]. In the context of HSV-1 infection, ISGylation of STING at multiple lysine residues (Lys224/236/289/345/370) has been detected. Among these, inhibition of ISGylation at Lys289 suppresses STING oligomerization and subsequently inhibits STING-mediated IFN-I activation [28]. In HIV, co-expression of the ISG15AA (ISGylation dysfunction mutant) with modified vaccinia virus Ankara (MVA-B) enhances HIV-specific CD8 ^+^ T cell responses and improves IFN-I secretion [110]. ISG15 also serves as an immune adjuvant when delivered via a DNA vector in a heterologous prime-boost regimen with an MVA-based recombinant virus expressing HIV-1 antigens Env/Gag-Pol-Nef (MVA-B) [ 110, 111].

Given that ISGylation plays a significant role in counteracting viral infections, focusing on specific substrates associated with this modification could present a promising approach for developing antiviral therapies. By enhancing or mimicking ISGylation in key proteins involved in viral replication or immune response, it may be possible to bolster the host’s defense mechanisms and inhibit viral propagation. This strategy could lead to the discovery of novel therapeutic targets and contribute to more effective treatments against a range of viral diseases.

#### ISGylation promotes viral infection by promoting viral nucleic acid replication and inhibiting immune signal transduction

ISGylation can also facilitate viral replication. For example, ISG15-deficient host cells exhibited reduced production of extracellular enveloped virus (EV) when infected with vaccinia virus (VACV) [112]. Additionally, ISGylation has been shown to inhibit antiviral signal transduction. For instance, adhesion and degranulation-protein adaptor protein (ADAP), an immune adaptor protein, was found to suppress RIG-1 ISGylation and promote the activation of IFN-I in macrophages, thereby countering RNA virus infection [113]. This dual functionality of ISGylation underscores the complexity of its role in viral interactions and immune responses.

### ISGylation also has a dual rule in antibacterial immunity

ISG15 also plays a dual role in combating bacterial infections. Similar to viral infections, bacterial infections induce ISG15 expression, which can, in turn, influence bacterial survival and dissemination by either enhancing bacterial clearance or promoting the antibacterial immune response.

ISGylation facilitates bacterial clearance. For example, ISG15 enhances the expression of pro-inflammatory cytokines, such as tumor necrosis factor-α (TNF-α) and interleukin-1β (IL-1β), in macrophages to eliminate *Streptococcus pneumoniae* ( *S. pn*). Autolysins are protein hydrolysis enzymes that are self-produced by bacteria and degrade bacterial cell walls. The autolysin of *S. pn* can inhibit ISG15 conjugation, blocking immune clearance by macrophages [114].

ISGylation promotes an antibacterial immune response. The E3 ligases HERC5 and mHERC6 mediate the ISGylation and degradation of phosphatase and tensin homolog (PTEN). The reduction of PTEN activates the PI3K-AKT signaling pathway, synthesizing pro-inflammatory cytokines in response to *Mycobacterium tuberculosis* *(M*. *tb)* infection, thereby facilitating the antibacterial immune response [36]. ISG15 and ISGylation are also upregulated upon *Listeria monocytogenes* ( *L. monocytogenes*) infection, which has been shown to confer protection against *L. monocytogenes* *in vivo* and *in vitro* [ 115, 116]. Furthermore, enhanced ISGylation has been reported to directly modify a subset of liver enzymes, promoting anti-infective immunity by temporarily reprogramming the organism’s metabolism after infection [117].

Conversely, ISGylation may increase host susceptibility to bacterial infections. Both IFN-I and ISG15 stimulate the replication of *M*. *tb* in murine cells, although mice lacking ISGylation exhibit reduced susceptibility compared to IFNAR-deficient mice [118].

Based on the studies mentioned, ISGylation is recognized as a significant factor in antibacterial immunity through its various mechanisms, such as enhancing pro-inflammatory cytokine production and promoting the degradation of proteins that regulate immune responses. However, its role in facilitating bacterial infection warrants further investigation. Understanding the dual nature of ISGylation could offer deeper insights into its impact on host-pathogen interactions and help to develop targeted therapeutic strategies that optimize antibacterial defenses while minimizing susceptibility to infections.

## ISGylation and Genomic Stability

DNA is an important macromolecule that carries biological genetic information. Its structural integrity and stability ensure that the body can carry out normal replication, transcription, translation, *etc*., and regulate the normal life activities of cells. The semi-conservative nature of DNA replication allows for stable inheritance in zygotic cells, ensuring that genetic information is accurately passed on. Additionally, the DNA damage response plays a vital role in safeguarding the DNA from damage caused by both internal factors and external stressors, thereby maintaining genomic stability and cellular health.

### DNA replication

DNA replication is a precise, semiconservative, enzyme-dependent process by DNA helicases, polymerases, ligases, *etc*. [ 119– 121]. Stalled replication forks are protected by factors such as breast cancer type 1 susceptibility protein (BRCA1), breast cancer type 2 susceptibility protein (BRCA2), Fanconi anaemia proteins ( *e.g*. FANCD2), DNA topoisomerase 2-binding protein 1(TOPBP1), Ataxia telangiectasia and Rad3-related protein (ATR), and replication protein A 32 kDa subunit (RPA32) from resection by exonucleases and endonucleases, including Exonuclease 1, DNA replication ATP-dependent helicase/nuclease DNA2, DNA structure-specific endonuclease MUS81, and the double-strand break repair protein MRE11 [ 122– 126]. Abnormal replication can also result in the formation of DNA bridges and micronuclei (MNs), which are prone to rupture during subsequent cell cycles [ 127– 130]. Then, the resection of stalled replication forks, DNA bridge breakage, and ruptured MNs release DNA into the cytosol, activating the DNA adaptor cGAS and the downstream innate immune IFN-I signaling pathway [ 122, 128, 131– 133].

**Figure 5:**
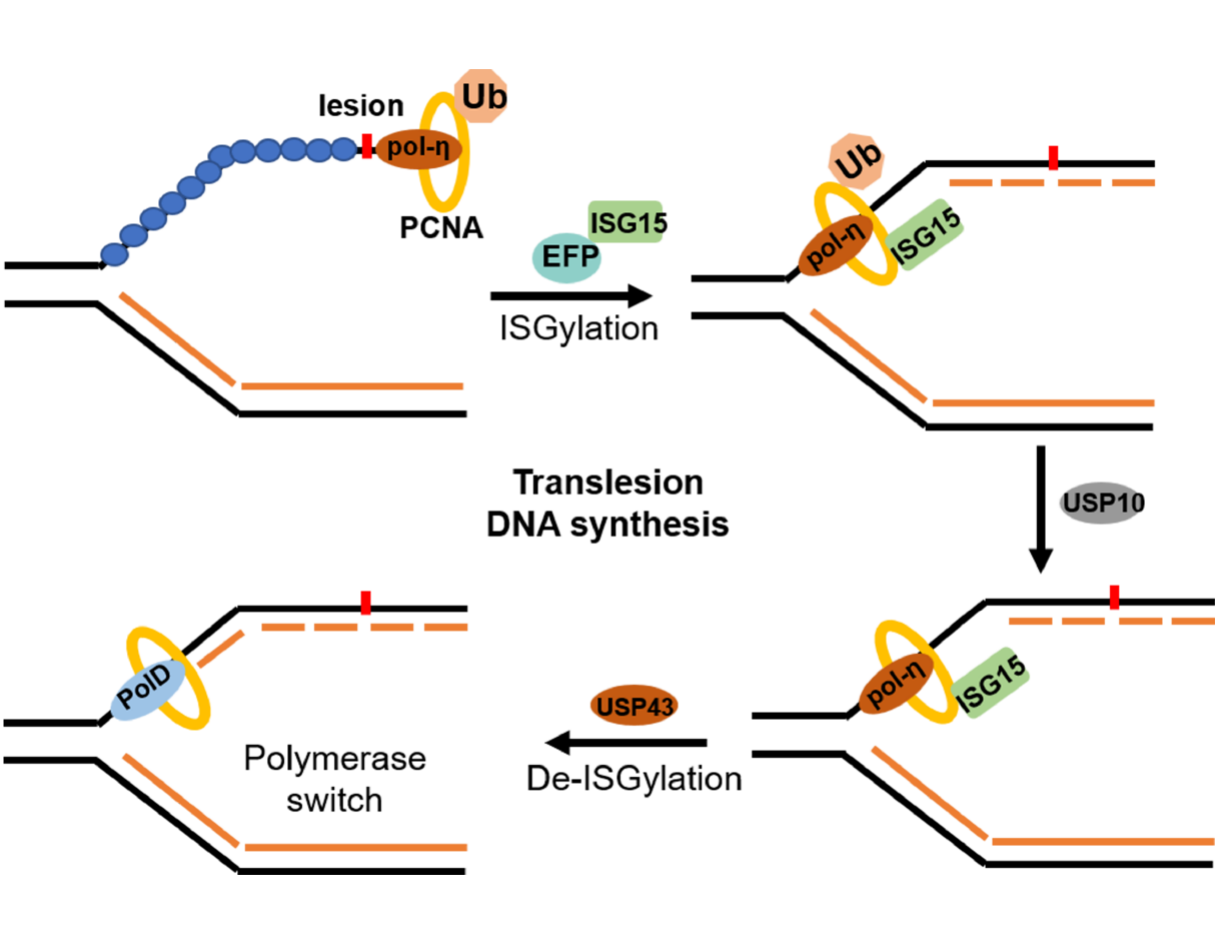
Figure 5 ISGylation promotes translesion DNA synthesis In response to replication lesions, PCNA monoubiquitination facilities PCNA ISGylation, initiating translesion DNA synthesis (TLS). Next, USP10 deubiquitinates PCNA and release polymerase-η. After TLS, USP43 catalyzes PCNA deISGylation and promotes fork restart. Red lightning symbol: DNA damage reagents.

When cells encounter DNA damage, the RAD6/18 complex monoubiquitinates proliferating cell nuclear antigen (PCNA) at Lys164, initiating translesion DNA synthesis (TLS) [ 134– 136]. ISG15 and ISGylation are critical in the restart of stalled replication forks ( Figure 5). Here, the E3 ligase EFP interacts with monoubiquitinated PCNA, facilitating its conjugation with ISG15. ISGylated PCNA then promotes USP10-mediated deubiquitination of PCNA, resulting in the release of polymerase-η. Following TLS, USP43 deISGylates PCNA, reloading DNA polymerases onto stalled replication forks and enabling the resumption of DNA replication [137].

DNA helicase RECQ-Like Type 1 (RECQ1) is another key helicase involved in restarting stalled replication forks. ISG15 interacts non-covalently with RECQ1, enhancing fork progression and reducing sensitivity to camptothecin (CPT)-induced fork slowdown, independently of ISGylation [ 138, 139]. This unregulated activity, however, may increase DNA damage and chromosomal aberrations [140]. ISG15 and ISGylation also protect nascent DNA from degradation. Disruption of ISG15/ISGylation results in MRE11-mediated degradation of nascent DNA. Conversely, treatment with IFN-β restores replication fork stability in breast cancer type 2 susceptibility protein (BRCA2)-deficient cells by upregulating ISG15/ISGylation and rescues the lethality of BRCA2-deficient mouse embryonic stem cells [141].

In summary, the mechanisms governing DNA replication are intricate and vital for maintaining genomic stability. ISG15 and ISGylation emerge as crucial players in this process, ensuring the proper functioning of stalled replication forks and protecting nascent DNA from degradation. Through their interactions with key proteins like PCNA and RECQ1, they facilitate the repair and resumption of DNA synthesis, reinforcing the resilience of cells against DNA damage. Understanding these processes highlights the importance of ISG15 in cellular responses to genotoxic stress and its potential implications in cancer biology and therapies, particularly in the context of BRCA1/2 deficiencies. This knowledge could pave the way for novel interventions aimed at enhancing genomic integrity and combating DNA repair-related diseases.

### The DNA damage response

Various exogenous factors, such as ultraviolet (UV) and ionizing radiation (IR) exposure, as well as endogenous stresses like replication and oxidative stress, can lead to DNA damage. Among these, double-strand breaks (DSBs) are the most severe, as a single unrepaired DSB can trigger apoptosis [142]. Failure to repair DNA damage promptly increases the risk of potentially disease-causing mutations. Indeed, disruption of genome integrity is closely associated with aging, cancer, and immunodeficiency disorders [143].

To counter these threats, cells have developed a sophisticated network known as the DNA damage response (DDR), which senses and responds to damage [144]. ISG15 is upregulated in response to UVC (a band of UV) radiation, IR, Camptothecin (CPT), and Aphidicolin (Aph), indicating its role in DNA damage response [ 131, 145– 147]. Defects in the MRE11/NBS1 complex increase ISG15 levels and ISGylation at replication forks and also induce cytosolic DNA formation, which activates the cGAS-STING pathway [146]. ISGylation suppresses cytosolic DNA formation, where several DDR proteins, such as TOP2A, FEN1, and VCP, are ISGylated and contribute to the stabilization of replication forks [146].

The tumor suppressor p53 is activated by DSBs and arrests the cell cycle at the G1/S phase boundary to facilitate the completion of DNA repair before DNA replication. If the repair fails, p53 initiates apoptosis, which eventually prevents oncogenesis [148]. ISG15 is implicated in the degradation of misfolded p53 mediated by USP18-mediated ISGylation, which subsequently disrupts HIV-1 virus replication and tumor progression in untransformed cells [148]. In transformed cells, SRC-mediated phosphorylation of p53 enhances its ISGylation, leading to increased degradation. Depletion of ISG15 increases DNA damage-mediated p53 activation, suggesting that ISG15 plays a crucial role in the DDR [33]. Moreover, the ISGylation of p53 mediated by E3 ligase EFP at K291/292 promotes p53 phosphorylation and acetylation and regulates downstream cell cycle arrest and apoptosis. These findings underscore the critical function of ISG15 in maintaining genome stability [32].

Cells have evolved a sophisticated DDR to detect and address these threats, with ISG15 playing a significant role in this process. Its upregulation in response to UV radiation, ionizing radiation, and other stressors highlights its involvement in the DDR. ISG15 facilitates the stabilization of replication forks by mediating the ISGylation of key DNA repair proteins. Additionally, the interaction between ISG15 and the tumor suppressor p53 further emphasizes its importance in regulating DNA repair, cell cycle arrest, and apoptosis. Ultimately, these findings underscore the essential role of ISG15 in maintaining genomic integrity and protecting against the onset of disease-related mutations. Understanding these pathways may provide valuable insights for therapeutic strategies aimed at enhancing DNA repair mechanisms in various disease contexts.

## Associations between ISGylation and Cancer

ISGylation has emerged as a crucial player in cancer biology, influencing tumor progression and response to therapy. This post-translational modification is primarily triggered by interferon signaling and plays a dual role in cancer: while it can enhance the immune response against tumors, it also contributes to oncogenic processes. Elevated levels of ISG15 and increased ISGylation have been associated with various malignancies, where they promote tumor cell survival, stemness, and metastatic potential. Moreover, ISGylation has been linked to the destabilization of critical tumor suppressors and the facilitation of resistance to chemotherapy and radiation therapy. Understanding the complex interplay between ISGylation and cancer will shed light on its potential as a biomarker for prognosis and therapy response, as well as a target for novel therapeutic strategies aiming to modulate this modification for better clinical outcomes.

### Implications of ISG15 in tumor development

For instance, the knockdown of Tripartite Motif Containing 29 ( *TRIM29*) suppresses stem cell-like features in pancreatic ductal adenocarcinomas (PDACs), an effect rescued by extracellular ISG15 via an autocrine mechanism [149]. ISG15 processing promotes macrophage polarization towards an M2-like phenotype, enhancing the migration and tumorigenicity of nasopharyngeal carcinoma (NPC) cells. ISG15 ^+^CD163 ^+^ macrophages inhibit the anti-tumor activity of CD8 ^+^ T cells in NPC [150]. Moreover, ISG15 facilitates the induction of E-cadherin on tumor-infiltrating dendritic cells (DCs), aiding tumor immune evasion [138]. High ISG15 expression in NPC is associated with increased recurrence, reduced survival rates, and resistance to radiation and cisplatin treatment [151]. Elevated ISG15 mRNA and protein levels correlate with lymphovascular invasion (LVI), histological grade, tumor size, HER2-enriched breast cancer subtypes, immune markers (CD8, FOXP3, CD68), and poor prognosis [152].

ISGylation has been widely implicated in promoting cancer progression by enhancing tumor malignancy, stemness, invasion, and therapy resistance. It facilitates oncogenic transformation, particularly through the conjugation of ISG15, which promotes Kirsten-Ras (Ki-Ras) transformation [153]. Furthermore, ISGylation contributes to the destabilization of crucial tumor suppressors, such as PTEN [154] and p53 [34], thereby diminishing their tumor-suppressive functions and promoting the growth of cancers like hepatocellular carcinoma.

ISGylation exacerbates intestinal inflammation and promotes colitis-associated colon cancer by antagonizing ubiquitin-dependent protein degradation, leading to ROS accumulation via interferon signaling [155]. ISGylation of SIRT1 facilitates lung cancer progression [62]. Endothelial Lipase (LIPG): ISGylation mediated by Deltex-3-like E3 ubiquitin ligase (DTX3L) prevents LIPG ubiquitin-dependent degradation, promoting tumourigenicity and metastasis in triple-negative breast cancer (TNBC) [24]. EGF Receptor (EGFR): ISGylation enhances EGFR recycling, sustaining Akt signaling and driving invasive breast cancer behavior [156]. Yes-associated Protein (YAP): ISGylation of YAP contributes to tumor formation in xenograft models [61]. In high-grade serous ovarian cancer (HGSOC), ISG15 enhances exocytosis and secretion of small extracellular vesicles (sEVs), promoting migration and invasion of cancer cells [157].

ISGylation also enhances tumor cell stemness and malignancy. In anaplastic thyroid carcinoma (ATC), abnormal ISG15 expression and ISGylation of Karyopherin Subunit Alpha 2 (KPNA2) are associated with heightened stemness and malignancy [41].

Besides promoting cancer progression, ISG15 and ISGylation also contribute to therapy resistance, serving as key mediators for resistance to chemotherapy and radiation therapy, particularly through their roles in the IFN-related DNA damage resistance signature (IRDS) [ 123, 158]. High levels of ISG15 expression are associated with resistance to radiation therapy and confer resistance to cisplatin in BRCA2-deficient breast cancer cells. Notably, inhibition of ISG15 sensitizes ATC and lung cancer cells to doxorubicin [ 41, 62, 141].

Interestingly, ISG15 also exhibits anti-tumour effects under specific conditions, largely through extracellular functions and immune regulation. For instance, extracellular free ISG15 has been shown to reduce breast tumor growth in nude mice by enhancing natural killer (NK) cell infiltration into tumors [159]. It also resulted in PD-L1 degradation. Overexpression of ISG15 promotes K48-linked ubiquitination and glycosylation-dependent proteasome degradation of PD-L1, improving the efficacy of PD-L1-targeted cancer immunotherapy [45].

Given such evidence, ISGylation is a double-edged sword in cancer biology. While it enhances tumor malignancy, invasion, and therapy resistance, its extracellular functions and role in PD-L1 degradation highlight its potential as a therapeutic target. A deeper understanding of ISGylation’s context-dependent effects is crucial for developing effective cancer therapies.

## ISGylation in Health

Aside from cancer, ISGylation has been implicated in various other diseases, including neurodegenerative, immune, and metabolic disorders. ISG15 and ISGylation have been identified as mechanisms underlying traumatic brain injury-induced neurodegeneration and proteinopathy in veterans [160]. Meanwhile, in Alzheimer’s disease, ISGylation of phosphorylated dynamin-related protein 1 (DRP1) affects mitochondrial function and dynamics [63]. Here, the transporter protein ABC subfamily G member 2 (ABCG2) is rapidly expressed, playing a protective role in epileptogenesis, a process by which a normal brain develops epilepsy via STAT2 and mTOR ISGylation [56].

In obesity-related type 2 diabetes mellitus, the E2 enzyme UbcH8 mediates STAT1 ISGylation, contributing to STAT1 activation and M1 macrophage polarisation in high-fat diet-induced obese mice, suggesting that targeting UbcH8 could be a therapeutic strategy [27]. ISG15 also influences heart failure development. The myofibrillar protein filamin-C (FLNC) is an ISGylation target. ISG15 regulates cardiac amino acid metabolism while promoting the accumulation of misfolded FLNC, which inhibits cardiomyocyte autophagy [52]. The ISGylation system also mitigates the upregulation of glycolysis induced by CVB infection or IFN treatment, reducing glucose demand and enhancing ATP production, which counters cardiac atrophy and dysfunction [106]. Based on these, inhibiting ISGylation, enhancing autophagy, or developing mitochondrial protectants could be potential therapeutic strategies.

LincRNA-ROR regulates HERC5-mediated p53 ISGylation, potentially contributing to liver fibrosis progression [35]. Defects in Gasdermin-E (GSDME) reverse CPS1 degradation mediated by deISGylation, alleviating ammonia accumulation and suggesting a promising therapeutic approach for acetaminophen-induced liver injury [64]. In acute kidney injury, inhibition of NOX4 ISGylation promotes its degradation, counteracting oxidative stress-induced damage [65].

Regenerative medicine is a multidisciplinary field focused on repairing, replacing, or regenerating damaged tissues and organs to restore normal function. It combines principles from biology, engineering, and medicine to develop innovative treatments for a wide range of diseases and injuries. Key strategies in regenerative medicine include stem cell therapy, tissue engineering, gene therapy, and the use of biomaterials. As a regenerative medicine, Peroxiredoxin II (Prx II) regulates MVB degradation and reduces exosome secretion from dermal mesenchymal stem cells (DMSCs) through TSG101 ISGylation, representing a potential target for therapeutic development [49].

## Conclusions and Future Perspectives

Much research has been conducted to understand how ISGylation functions in antiviral and antibacterial immunity, immune signaling, genome stability, and various human diseases. In-depth analyses of the molecular mechanisms of ISGylation are thus opening doors to new clinical strategies that intervene in this post-translational modification. ISGylation is particularly well-characterized in antiviral defense, where ISG15 conjugation to host and viral proteins restricts viral replication. This restriction occurs through inhibition of viral protein synthesis and assembly, enhancement of type I interferon signaling, and stabilization of antiviral proteins such as RIG-I and MxA [ 2, 26, 93]. The susceptibility of ISG15-deficient mice to viral infections highlights its critical role in antiviral immunity [108].

Additionally, ISGylation serves as a key link between innate and pathogen-specific immune responses. Emerging studies suggest that ISGylation contributes to antibacterial defense by promoting autophagy to degrade intracellular bacteria, aiding in phagosome maturation to eliminate pathogens such as *L. monocytogenes* and *M. tb*, and modulating inflammatory responses by stabilizing cytokine-signaling proteins [ 36, 115, 116].

In immune regulation, ISGylation influences critical signaling pathways that modulate immune responses. It enhances type I interferon signaling by stabilizing proteins such as STAT1, balances pro- and anti-inflammatory cytokine production, prevents immune overactivation, and maintains homeostasis [ 25– 27]. Notably, ISG15 deficiency is associated with hyperinflammatory responses, as observed in patients with ISG15-deficient autoinflammatory syndromes.

ISGylation also plays a dual role in tumor biology. By modifying proteins involved in DNA repair and cell signaling, ISGylation can either promote or suppress tumor growth, depending on the context. Elevated ISG15 levels are often linked to poor prognosis in cancers such as breast cancer. While ISGylation can enhance immune responses against tumors, excessive ISG15 expression may facilitate tumor progression by suppressing immune activity.

Beyond immunity and cancer, ISG15 functions as a regulatory molecule in cellular protein quality control. The interplay between ISGylation, ubiquitination, and SUMOylation is crucial for maintaining proteostasis. Pathogens have evolved mechanisms to evade ISGylation, with some expressing de-ISGylation enzymes that remove ISG15 from target proteins. ISGylation competes with ubiquitination and SUMOylation for lysine residues on target proteins and modulates signaling pathways by altering protein localization, stability, and activity. The combined effects of ISGylation and other PTMs ensure precise regulation of immune and cellular responses.

Clinically, ISGylation offers significant therapeutic potential. In infectious diseases, targeting ISGylation pathways could enhance antiviral and antibacterial immunity. In autoimmune disorders, modulating ISG15 levels may help address autoinflammatory conditions caused by ISGylation dysregulation. In cancer, ISG15 is being explored as both a biomarker and a therapeutic target.

**Figure 6:**
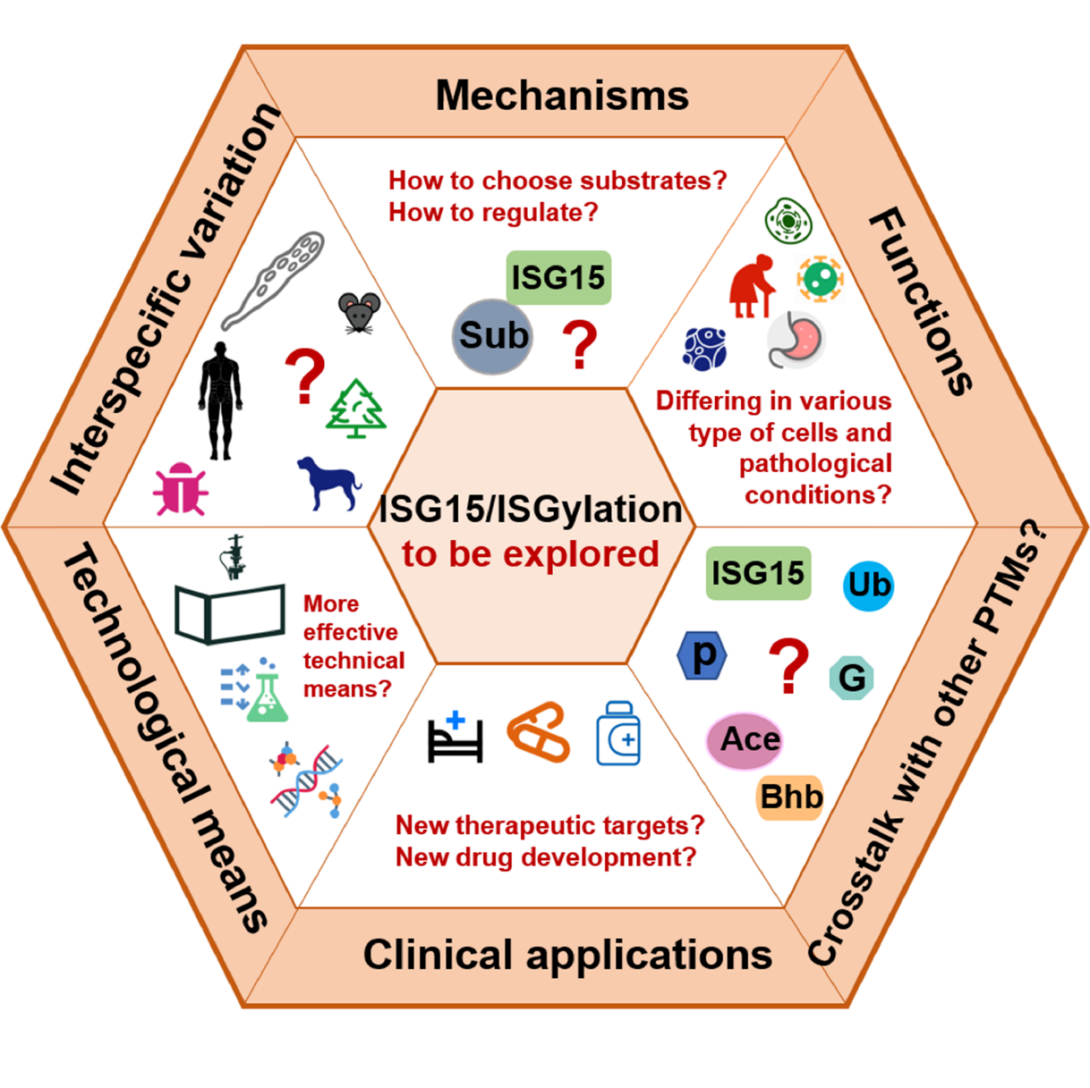
Figure 6 Research outlook on ISGylation Key avenues of investigation include determining the mechanisms by which ISG15 selects specific substrates and the signaling pathways involved in regulating ISGylation; exploring the functional roles of ISGylation in different cell types and pathological states; investigating the crosstalk between ISGylation and other post-translational modifications (PTMs), including ubiquitination, phosphorylation, acetylation, glycosylation, and β-hydroxybutyrylation; assessing the potential therapeutic applications of ISGylation, particularly its interplay with pathogen evasion strategies; evaluating the precision of current experimental tools in ISGylation research and developing more effective methodologies; and studying interspecies differences in the mechanisms and functions of ISGylation across organisms and their implications for interpreting research findings.

While research on ISGylation has expanded from its initial recognition as an antiviral mechanism to encompass roles in antibacterial immunity, immune regulation, cancer, and protein homeostasis, many questions remain. These include the identification of specific ISGylation targets and the mechanisms by which pathogens evade ISG15-mediated immunity. Continued studies are expected to uncover new therapeutic applications for modulating ISGylation in infectious diseases, cancer, and inflammatory disorders. Future research could now delve deeper into the following areas ( Figure 6).

1) Mechanism of ISGylation: Investigate how ISG15 selects substrates among numerous proteins. That would help us to better understand the functions and mechanisms, discover potential associations with diseases, develop specific drugs, and even identify potential biomarkers.

2) Regulation of ISGylation: Identify the signaling pathways and molecules that regulate ISGylation and how different immune responses are coordinated.

3) Functional diversity of ISGylation: Assess whether the function of ISGylation varies across different cell types ( *e.g*., immune cells, epithelial cells) and in different pathological states ( *e.g*., infection, cancer). By evaluating the function of ISGylation in different cell types and pathological conditions, potential therapeutic targets can be identified to develop targeted therapies for specific cells or disease states. Changes in ISGylation in different conditions may become new biomarkers for early disease detection, prognostic assessment, or therapeutic response monitoring. The diversity of ISGylation provides a wealth of information for systems biology studies to help build more comprehensive models of cell signaling networks and understand how different cell states and environments collectively affect protein function.

4) Interactions with other modifications: Explore how ISGylation interacts with other PTMs ( *e.g*., acylation, glycosylation) and determine whether these interactions follow any unknown principles ( *e.g*., cell-specific regulation?) affecting cell function. By studying the interaction between PTMs, a complex regulatory network can be constructed that will help scientists better understand intracellular signaling and regulatory mechanisms. The reciprocal regulation of PTMs can help cells adapt rapidly to environmental changes. For example, in the stress response, by adjusting the relative levels of multiple modifications, cells can achieve rapid functional adaptation.

5) Clinical relevance: Define the specific roles of ISGylation in various diseases ( *e.g*., mental illnesses and psychiatric disorders, anti-aging, medical cosmetology, *etc*) and assess its potential as a therapeutic target. With people’s pursuit of a better life and spiritual health, concern for physical and mental health, and the increasingly rapid development of the medical aesthetic industry. The investigation of ISGylation from these perspectives may promote industry reform to bring more therapeutic and economic benefits. Understanding the interplay between ISGylation and pathogen evasion strategies is also crucial for guiding novel therapies.

6) Refinement of research methods: For more efficient and precise research on the functions and mechanisms, evaluate whether current techniques used to study ISGylation ( *e.g*., mass spectrometry, antibody detection) possess sufficient sensitivity and specificity or whether new techniques are needed to probe ISGylation more deeply. Optimizing experimental design can improve the reproducibility and reliability of experiments to generate more meaningful results.

7) Interspecies differences: Explore variations in the mechanisms and functions of ISGylation across different species ( *e.g*., mouse vs. human) and investigate how these differences impact the interpretation of findings. There is no conflicting data at present.

Future research in these areas will deepen our understanding of the biological roles and therapeutic potential of ISGylation, offering novel strategies for treating infectious diseases, cancer, and inflammatory disorders.
